# Dataset of 16S rRNA and alkB genes in hydrocarbon polluted soils of Kuwait as revealed by Pyrosequencing

**DOI:** 10.1016/j.dib.2022.108434

**Published:** 2022-07-03

**Authors:** Sabah Al-Momin, Nazima Habibi, Rita Rahmeh

**Affiliations:** Biotechnology Program, Environment and Life Sciences Research Center, Kuwait Institute for Scientific Research, PO Box 24885 Safat, 13109, Kuwait

**Keywords:** Hydrocarbon degrading bacteria, Next generation sequencing, Bioremediation, Contaminated soil

## Abstract

The data in this article was generated by high throughput sequencing of moderately hydrocarbon polluted sites (S1 and S2) and a heavily polluted site (S3) in Kuwait. Deoxyribonucleic acid (DNA) extracted from each site was subjected to polymerase chain reaction (PCR) amplification employing conserved primers of 16S rRNA and alkB genes. Unique Molecular Identifiers (MID) tags were added to individual samples prior to pooling and sequencing on a Roche GS FLX platform using Pyrosequencing Titanium Chemistry. Raw sff files were deposited to the public repository of National Centre for Biotechnology Information (NCBI) under accession no PRJNA816075. The sff files were clipped according to the MID tags and converted to fasta format. 16S rRNA gene sequences were aligned against the SILVA database. The predominant genera at S1 and S2 was *Alkanindiges* whereas *Alcanivorax,* was highly abundant at S3. *Alkanindiges* have been found to play a key role in hydrocarbon degradation and *Alcanivorax* genus is known for its hydrocarbon degrading capability. The alk B gene sequences were subjected to blastx. The diversity of alkB gene was higher in S3 as compared to S1 and S2. These findings may open the way to the use of the genera *Alkanindiges* and *Alcanivorax* in the rehabilitation of hydrocarbon-contaminated sites in hot, arid climates. The isolation of these microorganisms and the design of bioaugmentation procedures specific to the dry climate could be a key step towards the restoration of hydrocarbon contaminated soils.

## Specifications Table

**Table:** 

Subject	Environmental Sciences
Specific subject area	Genomics
Type of data	Tables, figures, raw sequencing reads, OTU file
How the data were acquired	Pyrosequencing was conducted on a Roche 454 FLX instrument using the Titanium chemistry
Data format	Raw data sff file
Description of data collection	DNA was extracted from the soil samples and amplification of both the 16S rRNA and alkB genes was done using conserved primers. The amplicons were sequenced on a Roche platform and standard bioinformatics pipelines were used for subsequent analysis.
Data source location	• Institution: Kuwait Institute for Scientific Research • City/Town/Region: Kuwait • Country: Kuwait • Latitude and longitude (29° 18′ 50.67″ N; 47° 29′ 30.30″ E)
Data accessibility	Repository name: National Centre for Biotechnology Information and figshareData identification number: PRJNA816075; https://www.ncbi.nlm.nih.gov/sra/?term=PRJNA816075Direct URL to data: https://figshare.com/s/69fd83aa71333bdd1c0c

## Value of the Data

• Data are useful for the bioremediation of hydrocarbon polluted soils generated by oil producing companies.
• This data will also be interesting for environmentalists with concern on soil pollution by oil.
• The complete dataset can be used to design a series of quantitative polymerase chain (qPCR) reactions assays than can be used as monitoring tool for evaluating the effectiveness of bioremediation strategies.
• The hydrocarbon degrading potential of the genera identified in the present dataset can be exploited further.

## 1 Data Description

**Fig. 1:**
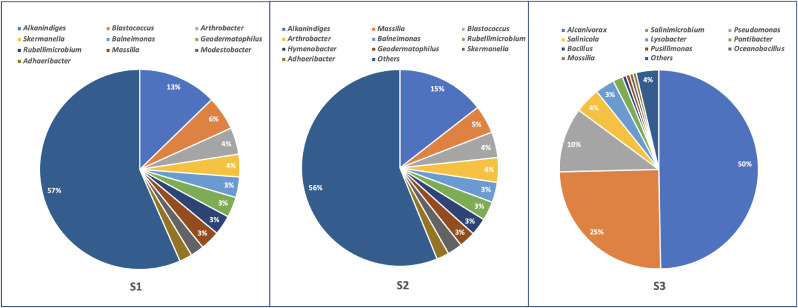
Relative abundance of top ten bacterial genera associated with hydrocarbon polluted soil samples collected from Kuwait.

The hydrocarbon pollution is a challenge in oil producing countries like Kuwait and the top-soil is the most exposed area to the oil spills. The influence of the top-soil contamination is more pronounced on the terrestrial life forms. The aim of this dataset was to determine whether the bacterial population diversity of the hydrocarbon polluted top-soil in the collected soil samples (*n* = 3) was reflected in the diversity of a key functional gene crucial to the biodegradation of hydrocarbons present in soils. The soils were collected from a hydrocarbon-polluted site. The samples S1 and S2 originate from oil-contaminated soil located at *(28° 58′ 35.6412" N; 48° 09′ 38.9099" E and the S3 from 28° 58′ 43.4759" N; 48° 10′ 07.8186" E* . The samples were collected at 0-20 cm depth from the surface by using a sterilized stainless steel shovel as per the standard method reported elsewhere [1]. The samples were passed through a 1-mm sieve before the TPHs concentration was determined and the DNA was extracted for sequencing. The TPH concentration in both S1 and S2 samples is above 1% and in S3 sample is above 5%. A total of 54904 individual 16S rRNA sequences were obtained from the 3 samples. 25687 were obtained from sample 1, 15422 from sample 2 and 13795 from sample 3. The distribution of the 16S rRNA read lengths for each sample was as expected with the mean read length just over 400 bases for each sample. The various individual good quality sequence reads for each sample were then clustered into groups where each group contains identical sequences. A total of 487 operational taxonomic units (OTUs) were obtained for sample 1 with only 200 showing a relative abundance (RA) above 0.04. For sample 2, total 436 OTUs were obtained and 157 had a RA above 0.04%. Sample 3 had least number of OTUs (99) with merely 27 with RA > 0.04%. Classification of OTUs was performed up to genera level. The top ten genera in the three soil types are shown in Fig. 1. Samples S1 and S2 had almost similar taxonomic profile whereas S3 exhibited different taxonomies. The predominant bacterial groups present in sample 3 were mostly halophiles (salt tolerant). At S1 and S2, *Alkanindiges* topped the list whereas *Alcanivorax* was highly abundant at S3 (Fig. 1). These genera were recognized as hydrocarbon degrading bacterium.

**Fig. 2:**
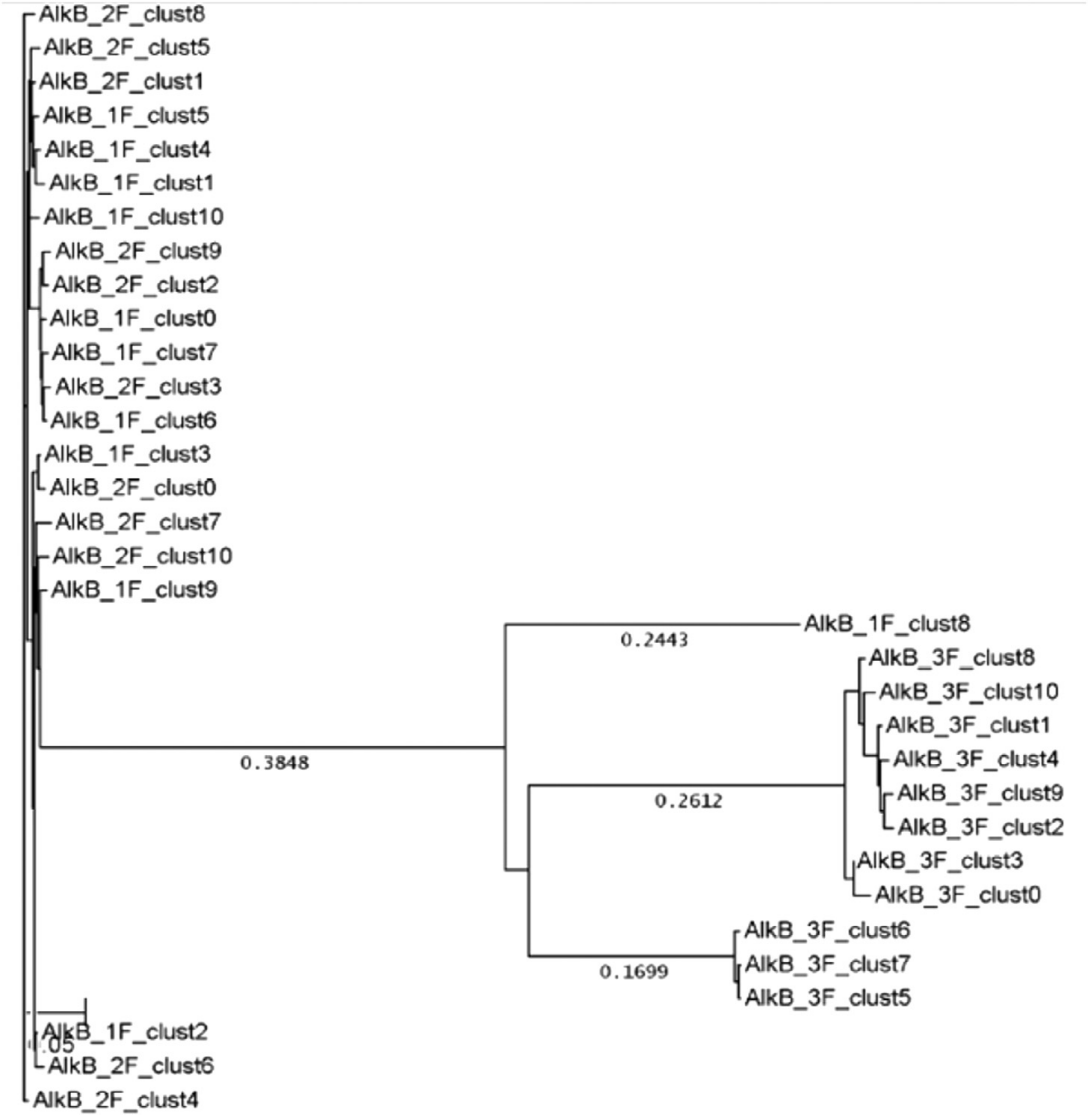
A neighbour joining tree of alkB gene sequences. The Tajimas Nei Distance between the sequences were calculated.

A total of 30491 individual alkB sequences were obtained from the 3 samples. 7915 were obtained from sample 1, 14906 from sample 2 and 7670 from sample 3. A total of 2403 groups were obtained for sample 1 with only 74 groups containing 10 or more sequences. The equivalent figures for sample 2 are 4246 total groups and 148 with 10 or more sequences and for sample 3 are 4076 total groups with 74 containing 10 or more sequences. The majority of the groups from all three samples contained less than 5 sequences with a significant number containing only one sequence. The top 10 sequences obtained from each sample were inserted into the DSGene software package and a ClustalW alignment was performed using the default parameter settings. Clustering of alkB gene based on their abundances distributed the samples into two groups namely A (S1 and S2) and B (S3) (Fig. 2).

The Shannon's diversity index is a measure of diversity that combines richness and the relative abundances. This index for the first 51 16S rRNA and alkB groups is shown in Table 1. The population diversity of sample 3, based on the 16S rRNA, is much less than that calculated for samples 1 and 2. Samples 1 and 2 are equally diverse based on the 16S rRNA data. Conversely, sample 3 is more diverse than samples 1 and 2 based on the alkB data set.

**Table 1:** Shannon's diversity indices of soil samples collected from hydrocarbon polluted sites in Kuwait.

	16S rRNA			alkB		
	S1	S2	S3	S1	S2	S3
No. of Groups	51	51	51	51	51	51
Total No. of Individuals	19716	11893	13695	4130	7295	2261
Shannon Diversity Index (H)	3.45	3.43	1.61	2.37	3.38	3.57

HTS methodologies targeting both a bacterial taxonomy gene (16S rRNA) and a functional gene (alkB) demonstrated significant differences between the samples provided. Samples 1 and 2 were similar to each other but different from sample 3 when compared at the taxonomic level (16S rRNA) or functional gene (alkB) gene levels. These significant differences may be due to the level of hydrocarbon contamination in the soil. At the population (taxonomic) level sample 3 was much less diverse than samples 1 and 2, which were similar. This may indicate that the salt environment constrains the development of a diverse bacterial population.

## 2 Experimental Design, Materials and Methods

### 2.1 DNA Isolation and High Throughput Sequencing

DNA was extracted from the soils using the MO BIO PowerSoil DNA Isolation Kit (MO BIO Laboratories, West Carlsbad, CA, USA). In total 250mg of soil sample was used for DNA isolation as per the manufacturer's instructions. The concentration and purity of the extracted DNA was determined using UV spectrophotometry (A260/A280 ratios) Conserved primers (16S rRNA forward primer: CAGCMGCCGCGGTAATWC 16S rRNA reverse primer: CCGTCAATTCMTTTRAGTTT; alkB forward primer: AAYACNGCNCAYGARCTNGGNCAYAA, alkB reverse primer: GCRTGRTGRTCNGARTGNCGYTG) were used to amplify a region of 400 and 550 bp regions of the 16S rRNA and alkB genes respectively [2,3]. In brief, 10 pg of DNA, 1 × Taq buffer, 200 μM of deoxynucleoside triphosphate (DNTPs), 25 pMol of primers, and 1.25U of Taq polymerase were added to make a reaction volume of 50 µL. PCR was run for 21 cycles of denaturation at 94°C (1 min), annealing 45°C (1 min), and extension 72°C (2 min). PCR amplification was visualized on an Agilent 2100 Bioanalyzer [4]. The PCR amplicons were tagged with unique MID sequences and pooled for clonal amplification by emulsion-based PCR and then sequenced using Pyrosequencing Titanium chemistry on a Roche GS FLX platform.

### 2.2 Bioinformatics Analysis

The raw .sff files checked for quality scores and processed for removal of MID tags. Clean sequences were submitted to cdhit-v4.5.4-2011-05-12 [5]. Each tag pool was clustered separately using a sequence identity threshold of 98.00%. The bidirectional sequencing reads of 16S amplicon samples were clustered (98% identity). The representative reads were then aligned to the SILVA database [6]. All Cluster-Representatives of alkB amplicon samples were blasted against a selected subset of alkB genes extracted from NCBI home page (alkB_ncbi_genes.fasta, 7972 sequences). The top 10 sequences obtained from each sample were inserted into the DSGene software package [7] and a ClustalW alignment performed using the default parameter settings for the alkB gene [8]. A phylogenetic tree was created using the Neighbour Joining Tree Building Method and Tajima-Nei Distance default settings [7]. The Shannon Diversity index (H) was calculated for the first 51 16S rRNA and alkB groups in each of the three samples [9].

## Ethics Statements

Not applicable.

## CRediT authorship contribution statement

**Sabah Al-Momin:** Conceptualization, Writing – original draft, Writing – review & editing, Supervision, Resources, Investigation, Project administration. **Nazima Habibi:** Conceptualization, Writing – original draft, Writing – review & editing, Formal analysis, Software, Visualization. **Rita Rahmeh:** Conceptualization, Writing – original draft, Writing – review & editing, Methodology, Investigation.

## Declaration of Competing Interest

The authors declare that they have no known competing financial interests or personal relationships that could have appeared to influence the work reported in this paper.
